# Thin-Film Transistors

**DOI:** 10.3390/membranes12040411

**Published:** 2022-04-09

**Authors:** Feng-Tso Chien, Yu-Wei Chang, Jo-Chin Liu

**Affiliations:** Department of Electronic Engineering, Feng Chia University, Taichung 407, Taiwan; j96590@gmail.com (Y.-W.C.); julia09281996@gmail.com (J.-C.L.)

Thin film transistors (TFTs) are key components used in a variety of fields such as solar cell, active-matrix liquid crystal displays (AM-LCDs), pixel switches, peripheral driver circuit and flexible electronics. With many studies and technological breakthroughs in TFTs, the display technology of active array liquid crystals using these devices as switching elements has matured. To achieve high performance color saturation, response time, lifespan, and flexible use of the panel, which are highly desirable on the market, TFTs are of particular interest in studies of various semiconductor materials, gate dielectrics, improved device structure, core processing and device simulation.

This Special Issue, entitled “Thin-Film Transistors”, for the journal *Membranes,* aims to discuss the significant recent progress in TFTs. Twelve research articles contribute to this Special Issue. The selected topics include metal oxide and semiconductor materials, device characteristics improvement, fabrication process, theory discussion, device structure simulation, and integration circuit implementation.

For the improvement of metal oxide semiconductor materials and device characteristics, metal oxide semiconductors have higher electron mobility than amorphous silicon to improve display panel resolution. A more powerful display can be achieved in terms of refresh rate, greater efficiency in power consumption, integration applications such as flexible displays or integrated circuits. With an Ar-O_2_ mixed plasma treatment and rapid thermal annealing, dual gate (DG) indium–gallium–zinc oxide (IGZO) TFTs can decrease the oxygen vacancy density in the IGZO thin film and demonstrate a greater accumulation of conduction electrons caused by modulation of the carrier transportation path in a DG structure [1], therefore improving device performance. Indium oxide has high transmittance features, and tin oxide has strong conductivity. Indium tin oxide ITO TFTs have attracted great attention in the field of displays and low-cost integrated circuits. The proper channel thickness on ITO TFT performance and positive bias stress stability are examined in [2] to achieve better device performance. It is found that the thickness of ITO dominates the content of oxygen defects and therefore synthetically affect the device characteristics by trap states and carrier concentration. Two-dimensional (2D) crystals have been suggested as a class of materials that may impact future electronic technologies. Black phosphorus is a single elemental 2D material with a sizable band gap and remarkable high hole mobility that is suitable for developing future nanoelectronic applications. Dewu Yue et al. [3] reveal that ambipolar black phosphorus devices fabricated by capping with transparent hexagonal boron nitride offers a feasible approach to implement digital circuits and high photoresponse BP photodetectors.

TFT characteristics can be improved by different process methods. A steep subthreshold swing (SS) can be achieved with plasma oxidation on a SiNx gate dielectric for InGaZnO TFTs by reducing the amount of oxygen vacancy near the interface between the SiN_x_ and InGaZnO layer [4]. A Zirconium-doped Mg_x_Zn_1−x_O (Zr-doped MZO) mixed-oxide film by radio-frequency magnetron sputtering to reduce resistance and improve the electron mobility of MZO films is proposed in [5]. The dielectric layer is also an important core material of the TFTs. High relative dielectric constant (high-k) and a wide band gap material can be achieved by using multiple metals to increase the entropy of the structure. Four metal oxides, zirconium–yttrium–aluminum–magnesium-oxide (ZYAMO), high-k dielectric layers were prepared using the solution method to reduce device leakage and improve the ability to store charges, as discussed in [6]. 

Channel traps and structural disorder in an amorphous oxide semiconductor (AOS) affect TFT’s performance. An empirical modeling procedure to capture the gate bias dependency of In-Ga-Zn-O (IGZO) AOS TFTs while considering contact resistance and disorder effect is described and derived in [7], which is helpful to the development of an accurate compact TFT model. The hysteresis in TFT is another issue affecting circuit operation. It is believed that holes trapped in the dielectric are the main source of hysteresis. Improved hysteresis of the polycrystalline SnO_2_ TFTs by various curing and a fixed annealing temperature to analyze and understand hysteresis behaviors and its application to the circuits is studied in [8]. Nanowire junctionless TFTs also demonstrate high potential in circuit application, owing to their small number of defects, high-density integration, and flexible dimension design. Poly-Si nanowire TFTs with external connection of a BiFeO_3_ capacitor is studied to improve device characteristics on on-current and SS [9]. Moreover, a small hysteresis effect in this structure is achieved. 

Technology Computer-Aided Design (TCAD) also provides a powerful way to predict device performance. High bandgap materials use TCAD to construct a suitable process and device structure and is a low-cost way to set up a pre-study procedure. A nitrogen ion implantation, along with an annealing process converting thin film normally on AlGaN/GaN structures into normally off devices is simulated and discussed in [10]. TCAD simulation also help us to understand the device’s physical operation mechanism. Offset, LDD (lightly doped drain), GOLDD (gate overlapped lightly doped drain) and RSD (raised source drain) structures are common designs to reduce a device’s electric field and improve the kink effect and leakage current. TCAD helps researchers to design a new device frame. An RSD and vertical LDD concept in TFTs are discussed in regard to different structure simulations [11]. The performance of more complicated thin film transistors can also be predicted by the TCAD simulation procedure. 

For circuit application, most organic semiconductors exhibit p-type properties, unlike 2D materials and most oxide semiconductors; those are almost n-type devices. P-type structures are very important for the fabrication of CMOS inverters. Furthermore, their simple synthesis process allows them to be fabricated on flexible devices. Carbon nanotubes (CNT) used in TFTs have attracted great attention for their printable synthesis method and p-type semiconductor characteristics. However, CNT is still limited due to inadequate air stability and limited tunability. Gunhoo Woo et al. [12] discuss the emerging materials for thin film transistors and the methods of using hybrid thin film material combinations for complementary integration circuit implementation in detail. A capping process with transparent hexagonal boron nitride offers a feasible approach to implement both n and p type semiconductors [3]. Those provide some ways to achieve digital circuits by TFTs.

In conclusion, the above contributions show the recent developments in the area “Thin Film Transistor” research. This Special Issue includes the emerging materials and related process technologies to improve device performance, and the need for further development in the TFTs field. 
